# Overexpression of mineralocorticoid receptors does not affect memory and anxiety-like behavior in female mice

**DOI:** 10.3389/fnbeh.2015.00182

**Published:** 2015-07-14

**Authors:** Sofia Kanatsou, Laura E. Kuil, Marit Arp, Melly S. Oitzl, Anjanette P. Harris, Jonathan R. Seckl, Harm J. Krugers, Marian Joels

**Affiliations:** ^1^Department of Translational Neuroscience, Brain Center Rudolf Magnus, University Medical Center UtrechtUtrecht, Netherlands; ^2^Center for Neuroscience, Swammerdam Institute for Life Sciences, University of AmsterdamAmsterdam, Netherlands; ^3^Endocrinology Unit, Centre for Cardiovascular Science, Queen’s Medical Research Institute, The University of EdinburghEdinburgh, UK

**Keywords:** fear, memory, mineralocorticoid receptor, hippocampus, sex, anxiety

## Abstract

Mineralocorticoid receptors (MRs) have been implicated in behavioral adaptation and learning and memory. Since—at least in humans—MR function seems to be sex-dependent, we examined the behavioral relevance of MR in female mice exhibiting transgenic MR overexpression in the forebrain. Transgenic MR overexpression did not affect contextual fear memory or cued fear learning and memory. Moreover, MR overexpressing and control mice discriminated equally well between fear responses in a combined cue and context fear conditioning paradigm. Also context-memory in an object recognition task was unaffected in MR overexpressing mice. We conclude that MR overexpression in female animals does not affect fear conditioned responses and object recognition memory.

## Introduction

Exposure to stressful experiences activates the Hypothalamus-Pituitary-Adrenal (HPA)-axis, which—among other things—results in elevated plasma levels of corticosteroid hormones (corticosterone in rodents, cortisol in humans; Joels and Baram, 2009). Corticosteroids bind to two types of corticosteroid receptors: mineralocorticoid receptors (MRs) and glucocorticoid receptors (GRs), which differ in their localization in the brain and affinity for corticosterone (Reul and de Kloet, 1985; de Kloet et al., 2005). Both MRs and GRs can exert slow genomic actions on cellular function, but recent studies have demonstrated that activation of these receptors can also activate fast membrane receptor mediated non-genomic pathways (Di et al., 2003; Karst et al., 2005, 2010; Groc et al., 2008; Groeneweg et al., 2011).

In male rodents, corticosterone acting via MRs facilitates spatial learning (Berger et al., 2006; Lai et al., 2007), reduces anxiety (Lai et al., 2007; Rozeboom et al., 2007) and improves the formation of contextual fear (Zhou et al., 2011). Moreover, MR activation regulates the selection of appropriate behavioral strategies in the face of stress, favoring a switch from hippocampus-dependent to striatal learning strategies (Schwabe et al., 2010, 2013). Overall, these studies in rodents suggest that MR activation favors behavioral adaptation to stressful events.

Also in humans, MRs are important for neuroendocrine function and behavioral adaptation (DeRijk et al., 2006; Otte et al., 2015). Two single-nucleotide polymorphisms (SNPs) of the human MR gene (−*2G/C* and *I180V*) have been associated with variability in MR functionality. Specifically, a common haplotype involving these SNPs (MR-2C/MRI180) was associated with high MR expression and trans-activational activity *in vitro* (van Leeuwen et al., 2011). Individuals carrying this haplotype also displayed high salivary and plasma cortisol responses in a psychosocial stress situation (van Leeuwen et al., 2011). Homozygous female but not male carriers of haplotype 2 were found to have higher dispositional optimism, fewer thoughts of hopelessness and a lower risk on major depression (Klok et al., 2011).

Thus, in general MRs seem to enhance behavioral adaptation to stressful events, facilitate (fear) learning and memory, and promote resilience to stressful events (de Kloet et al., 2005). However, most studies that specifically investigated learning and memory in rodents so far focused on the MR in males; relatively little is known about the effect of (enhanced) MR function in females (ter Horst et al., 2013; Arp et al., 2014). Since sex-differences in MR function appear to exist in humans and rodents, we examined in this study whether forebrain-specific overexpression of MRs in female mice affects contextual memory formation, emotional memory formation and anxiety.

## Materials and Methods

### Animals

All mice used in our experiments were bred in-house. In each breeding cage, two wild type C57Bl6 female mice (Harlan, The Netherlands) were housed with one MR-transgenic (MR-tg) male mouse (Lai et al., 2007) for 1 week. Subsequently, the male mice were removed and the female mice were left undisturbed until day 18 of their pregnancy. From this point in time, the female mice were individually housed until they gave birth. We preferred to use wild type rather than MR-tg dams, to keep maternal care as comparable as possible to earlier studies in C57Bl6 mice. At postnatal day (PND) 23, all pups were weaned, genotyped and female pups with identical genotypes were housed four per cage. Mice were left undisturbed (except for cage cleaning once a week) until testing, when they were 3–3.5 months of age.

Mice were kept in a temperature and humidity controlled facility (21.5–22°C with humidity between 40 and 60%) on a 12 h light/dark cycle (lights on at 8:00 a.m.) with food and water available *ad libitum*. All experiments were performed in accordance with the Dutch regulations for animal experiments (DED206).

### Body Weights and Basal Corticosterone Levels

The body weight of the mice was recorded before the initiation of behavioral testing. Two weeks after the completion of the behavioral test, mice were decapitated in the morning between 09:00 and 11:00 h and their trunk blood was collected in Ethylenediaminetetraacetic acid (EDTA)-covered capillary tubes (Sarstedt, Netherlands) to determine basal plasma corticosterone levels. These levels were measured in duplicate via a radioimmunoassay kit according to the manufacturer’s protocol (MP Biochemicals, Amsterdam, Netherlands).

### Behavior

We performed all behavioral tests during the light phase between 8:30 a.m. and 12:00 a.m. We used a different cohort of mice for each of the behavioral tests: (i) object-in-context recognition memory; (ii) contextual fear conditioning; (iii) cued fear conditioning; and (iv) combined cued and context conditioning. All four different cohorts of mice were first tested on the elevated plus maze at 3 months of age and 1 week later subjected to one of the behavioral tests listed above.

#### Elevated Plus Maze (EPM)

Mice were transferred from the housing room to the behavior testing room 30 min before the actual testing. The mouse was placed in the center of a plus maze (light gray plexiglass; open arms: length 36.5 cm, width 0.5 cm; closed arm: length 35.2 cm, width 0.5 cm, side walls: 15.0 cm; elevation poles: 58.5 cm, UGO BASILE S.r.l.—Italy). The maze was cleaned with 70% ethanol and dried thoroughly with paper tissue before the mouse was placed in the maze. At the start of the test, each mouse faced the same open arm. After 5 min of testing the mouse was removed from the plus maze and returned to its home cage. A camera above the maze was used to record the sessions. The videos were analyzed by Ethovision XT 6 (Noldus, Wageningen, Netherlands). We estimated the percentage of time spent in the open arm and the number of open arm entries; low values are considered to reflect anxiety-like behavior. The total distance moved in the maze (open and closed arms) was used as an indication of general locomotor activity.

#### Contextual Fear Conditioning

Contextual fear memory was examined as described before (Zhou et al., 2011). On day 1, the mouse was placed in a chamber (W × L × H: 25 cm × 25 cm × 30 cm) that had a stainless steel grid floor connected to a shock generator. After 3 min of free exploration a single foot shock of 0.4 mA was delivered for 2 s. 30 s later the mouse was removed from the chamber and returned to its home cage. On day 2, the mouse was placed in the same chamber for 3 min. The occurrence of freezing behavior [defined as no body movements except those related to breathing Zhou et al. (2009, 2010)] was checked and scored every 2 s on days 1 and 2. For analysis we calculated for each day the total time spent freezing as a percentage of the total duration of the test.

#### Cued Fear Conditioning

Cued fear conditioning was examined to assess amygdala-dependent (fear) memory formation. On day 1, the mouse was placed in a black chamber (W × L × H: 25 cm × 25 cm × 30 cm), that had a stainless steel grid floor connected to a shock generator (Context A). The mouse could freely explore this chamber for 3 min. Thereafter, a tone (100 dB, 2.8 kHz) was given, lasting 30 s; during the last 2 s the mouse received a single foot shock of 0.4 mA. Thirty seconds later, the mouse was returned to its home cage. Twenty-four hours later on day 2, the mouse was placed in another chamber with striped patterns on the walls and a smooth floor (Context B) and allowed to explore for 3 min. Thereafter, the same tone as on day 1 but without shock was delivered for 30 s; the mouse remained in this chamber for another 30 s before being returned to its home cage. Before each mouse was tested, chambers were cleaned: Context A with 70% ethanol and Context B with 1% acetic acid, providing also different smells to the environments. Freezing behavior of the mouse was scored every 2 s (see above). The analysis was performed by the same investigator as the one carrying out the behavioral test but blinded to the experimental groups during analysis.

#### Combined Cued and Context Conditioning

On day 1, the mouse was placed in a fear conditioning chamber (W × L × H: 25 cm × 25 cm × 30 cm) that was cleaned with 70% ethanol. The grid floor was made of stainless-steel rods and was connected to a shock generator (0.4 mA). A white light source and a camera were placed 20 cm above the chamber. An audio-speaker was connected to a tone generator and positioned on the wall of the chamber. During acquisition (day 1) the mouse was allowed to freely explore the chamber for 3 min. Then, the animal was exposed to six light/tone episodes (cue-on episodes; 20 s each) paired with a foot shock (0.4 mA) during the last 2 s. The interval between the light/tone + shock pairings was 1 min (the context, cue-off episode). Two minutes after the last pairing, mice were returned to their home cage. On day 3 (48 h later), the mouse was exposed to the same procedure as on day 1, but without shocks. Frequency and duration of freezing behavior was scored using Observer XT, Noldus, Wageningen, Netherlands. Freezing behavior was determined and quantified during cue on periods and cue off periods (i.e., after the foot shock) and was defined as no body movements except those related to respiration. This fear conditioning paradigm allowed a test of fear related behavior of the mice during alternating cue-on (light + tone together) and context (cue-off) episodes (Brinks et al., 2009) in the same experimental protocol, thereby enabling detection of generalization and specificity of fear.

#### Object-in-Context Recognition Memory

We tested the mice for place memory, a non-stressful behavioral task, to examine the influence of context on object recognition (Dix and Aggleton, 1999; Mumby et al., 2002; Eacott and Norman, 2004; O’Brien et al., 2006; Balderas et al., 2008; Spanswick and Sutherland, 2010; Spanswick and Dyck, 2012; Barsegyan et al., 2014). As context we used four blue-colored plastic boxes of identical measurements (W × L × H; 33 cm × 54 cm × 37 cm) with or without visual cues on the walls. The boxes contained bedding material and additional objects: blocks of Lego and/or small bottles.

Mice were tested on three subsequent days. On day 1, the mouse was placed for 10 min in a box with no wall cues and without objects. On day 2, the mouse was placed for 10 min in a box (context A) that had no cues on the walls but contained two identical objects, i.e., two blocks of Lego, placed in opposite corners. Thereafter, the mouse was placed for 10 min into another box (context B) with cues on the walls in the form of stripes and two (new) identical objects, i.e., 2 small bottles, placed in opposite corners. Between exposure to context A and context B, the mouse was returned to its own transport cage. On day 3 object-in-context recognition memory was tested by placing the mouse for 10 min in context B. Context B on day 3 contained one object which also belonged to context B on day 2 (i.e., familiar object to Context B), and one object which belonged to Context A on day 2 (i.e., unfamiliar object to context B, Figures 6A–C). We calculated the discrimination index (DI) on day 3 as a measure for object-in-context recognition memory. The DI was calculated as time spent with the novel object compared to the total exploration time of both objects [t_novel_ /(t_novel +_ t_familiar-_); Mumby et al., 2002; Akkerman et al., 2012]. All objects were cleaned thoroughly between tests, and placed at a 15 cm distance from the corners of the box. Fresh bedding material was added on top of the old and mixed between each session. Sniffing was scored as object-exploration behavior if the mouse displayed such behavior towards an object within a distance of 2 cm maximum. Climbing on top of or “watching” the objects from a (close) distance was not considered as sniffing behavior.

### Determination of the Cycle Stage

To take the cycle stage of the females into account, vaginal smears were taken immediately after each behavioral test using a smear loop (size 1 μl; Greiner Bio-one). Cells were transferred on a water drop on a glass microscope slide. Slides were allowed to dry overnight followed by Giemsa (Sigma) staining for 12 min.

### Statistical Analysis

Because all data were normally distributed, as determined by Shapiro-Wilk tests for normality (results not shown), we used parametric statistics. Statistical analyses were performed using Statistical Package for the Social Sciences (SPSS): two-tailed *t*-test when two means were compared; repeated-measures Analysis of Variance (ANOVA; when appropriate); and two-tailed paired *t*-test (averaged cue and context fear conditioning episodes).

We analyzed the results of the contextual fear conditioning and elevated plus maze task for each cycle stage, because the relatively large number of animals allowed subgroup analysis. For these tests we did not observe any consistent influence of the cycle in the behavioral performance (data not shown). In the other tasks subgroup analysis was not possible due to the rather low number of females in some stages of the cycle. We therefore grouped all stages in the results and tested the impact of cycle stage on behavioral performance with a General Linear Model analysis, including the cycle stage as a covariate.

A *p* < 0.05 was set as the level of significance (*) and a *p* < 0.10 was considered as a trend level (#). Data are presented as mean with standard error of the mean (SEM), with group size (n) indicated.

## Results

### Body Weights and Basal Corticosterone Levels

**Figure 1:**
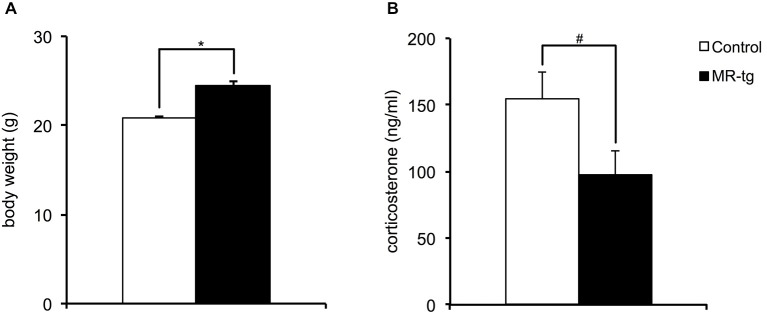
**Neuroendocrine parameters. (A)** Body weight measured before the initiation of behavioral testing revealed that female MR-tg mice weigh significantly more than control mice. *n* = 20–24 per group. **(B)** Basal a.m. plasma corticosterone levels measured 2 weeks after the behavioral paradigms showed that MR-tg mice show a trend towards significantly lower basal corticosterone levels than control female mice. *n* = 15–20 per group. *p < 0.05, ^#^trend, *p* < 0.10.

Body weight was measured from all animals before the start of the behavioral paradigms when animals were approximately 3.5 months of age. Female MR-tg mice were found to be significantly heavier in absolute body weight compared to control littermates (*t*_(69)_ = −7.92, *p* < 0.001; Figure 1A). MR-tg mice also displayed a trend towards significantly lower basal plasma corticosterone levels (*t*_(33)_ = 1.98, *p* = 0.055; Figure 1B).

### Elevated Plus Maze

**Figure 2:**
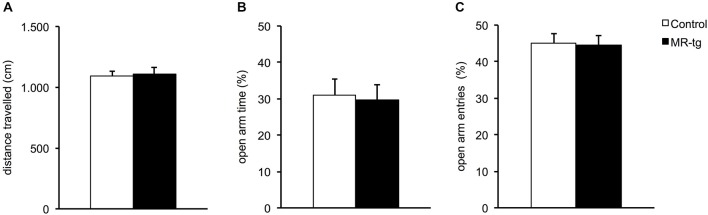
**Effects of MR overexpression in elevated plus maze. (A)** Forebrain MR overexpression did not alter locomotor activity in MR-tg vs. control female mice. **(B,C)** MR-tg and control mice exhibited no differences in anxiety-like behavior, as the percentage of time in the open arms **(B)** (out of all arm entries) and the percentage of open arm entries **(C)** were similar for both groups. *n* = 35–37 per group.

We tested control and MR-tg female mice at PND 90 with respect to frequency of open arm entries, percentage of time in the open arms and total distance the mice traveled in the elevated plus maze (EPM), for a total duration of 5 min (Figure 2). The frequency of open arm entries was similar for control and MR-tg mice (*t*_(70)_ = 0.19, *p* = 0.844). Control and MR-tg mice also spent a comparable amount of time in the open arms (*t*_(70)_ = 0.19, *p* = 0.844). Finally, the general locomotor activity was not different between control and MR-tg animals (*t* = 70 = −0.25, *p* = 0.799).

### Contextual Fear Conditioning

**Figure 3:**
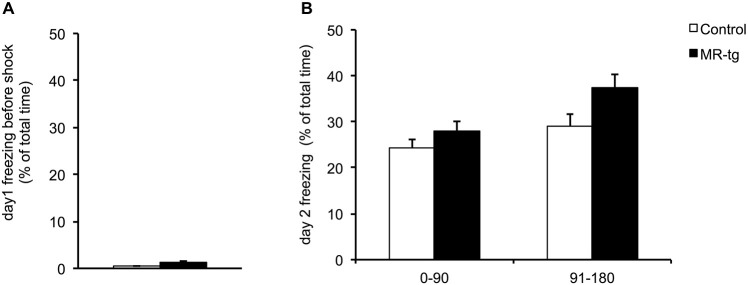
**Effects of MR overexpression on contextual fear conditioning. (A)** During training, female MR-tg and control mice exhibited no differences in freezing behavior in response to the context, measured for the total 3 min period of testing. **(B)** Twenty-four hours later, MR-tg mice show comparable freezing behavior compared to control mice, when tested over time (first 90 s compared to the last 90 s of time freezing). *n* = 25–30 per group.

During training and prior to the foot shock, MR-tg and control mice displayed little freezing behavior; the percentage of time was comparable for both groups (Figure 3A). During the retention test, twenty-four hours later, mice of both groups spent approximately 30% freezing of the total 3 min testing time (data not shown). Since MR is thought to be involved in early appraisal of fear, we distinguished between the first and second half of the observation period, as described before (Zhou et al., 2010). Dividing this period into two blocks of 1.5 min (Zhou et al., 2010) revealed that MR-tg and control mice displayed no differences in the percentage of time freezing (*F*_(1,52)_ = 0.086, *p* = 0.770; Figure 3B).

### Cued Fear Conditioning

**Figure 4:**
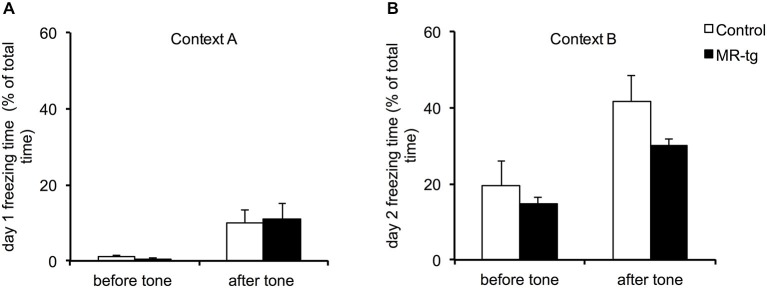
**Effects of MR overexpression on cue fear conditioning. (A)** During training, comparison between MR-tg and control mice revealed no differences in freezing behavior before as well as after the presence of the tone. *n* = 8 per group. **(B)** Twenty-four hours later, both MR-tg and control mice showed similar freezing behavior in response to the new context, when compared before and after the tone presentation. *n* = 8 per group.

During training, MR-tg and control mice displayed little freezing behavior before exposure to the tone and foot shock (Figure 4A). Exposure to the tone increased freezing behavior and freezing behavior was also increased after exposure to the foot shock, in a comparable manner for both groups (Figure 4A). Twenty-four hours later, both groups showed similar freezing levels both before and after the presentation of the cue exposure to the tone, now presented in a novel context (*F*_(1,22)_ = 1.087, *p* = 0.315; Figure 4B).

### Combined Cue and Context Conditioning

**Figure 5:**
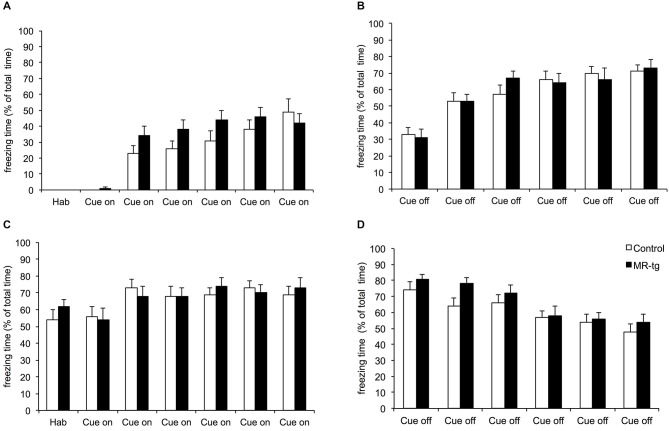
**Effects of MR overexpression on combined cue and context conditioning.** On the acquisition (day 1), animals were exposed to six tones followed by a foot shock. **(A)** Freezing behavior was scored during the tone (cue on) and after the tone (cue off) **(B)**. Forty-eight hours later mice were exposed to the same procedure as on day 1, but without shocks. Freezing behavior was scored during the tone (cue on) **(C)** and after the tone (cue off) **(D)**. No group differences were observed. *n* = 15–18 mice per group.

The combined cue and context fear conditioning paradigm allows detection of generalization and specificity of fear (Brinks et al., 2009). During acquisition (day 1) both MR-tg mice and wild type littermates increased freezing behavior during cue on and cue off periods (*F*_(11,341)_ = 76.761, *p* < 0.001), and always showed more freezing behavior during the cue off (i.e., after the footshock) when compared to the cue on period (Figures 5A,B), as described earlier for this particular paradigm (Brinks et al., 2008, 2009). No significant differences between MR-tg mice and control mice were seen. Fourty-eight hours after training, both control and MR-tg mice displayed freezing behavior during the cue on (Figure 5C) and cue off (Figure 5D) periods. Animals kept freezing in response to the tone (Figure 5C), while showing a decline in freezing behavior during the cue off periods (Figure 5D). As a result, animals started freezing less during cue off than during cue on after the fourth cue on exposure (*t*_(36)_ = −5.134, *p* < 0.0001; Figures 5C,D). No group differences were observed.

### Object-in-Context Recognition Memory

**Figure 6:**
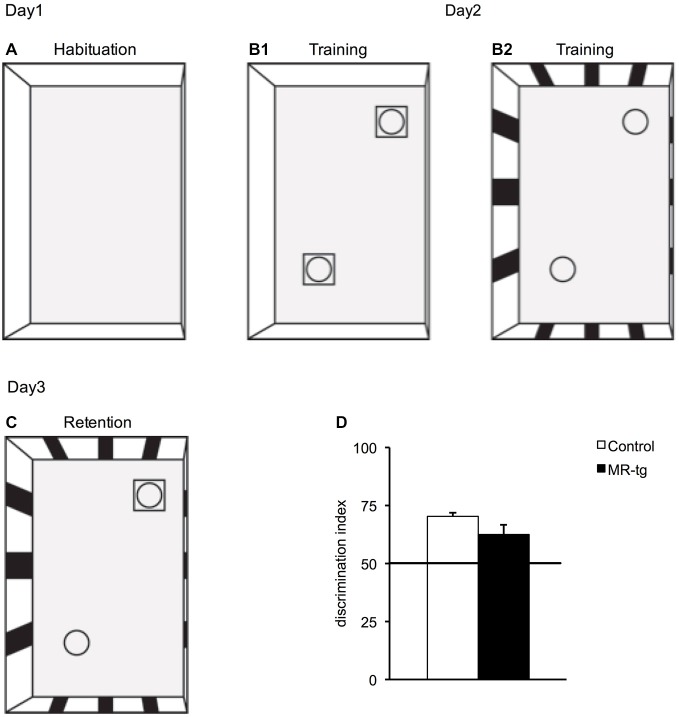
**Effects of MR overexpression in object-in context. (A–C)** Schematic representation indicating the setup of the object-in-context experimental paradigm: **(A)** On day1, mice were initially habituated in context A that had no objects. **(B1)** On day2, during training, mice were placed in the same context (context A) but with two identical objects and then placed in a novel context (context B) with two identical novel objects **(B2)**. **(C)** On day3, the mice were placed in the context B but with one object being replaced by an object from the first context. **(D)** MR-tg and control mice exhibited no differences when tested for recognition memory of a novel object in the context B, as the discrimination index (DI) of MR-tg mice was not significantly different from that of the control mice. *n* = 14 per group.

In the object-in-context memory test, mice displayed a preference for the unfamiliar object-context combination (i.e., mice displayed more exploration towards the object not previously explored in context B). Overall, the DI was higher than the chance level of 0.5 (Figure 6D). However, statistical analysis revealed no significant differences in the recognition memory between control and MR-tg female mice (*t*_(26)_ = 1.700, *p* = 0.101).

## Discussion

MRs have been implicated in orchestrating behavioral responses to stressful experiences (de Kloet et al., 1999; Schwabe et al., 2010). This was, for instance, evident by using pharmacological and transgenic manipulations in mice (Schwabe et al., 2010; Arp et al., 2014). Interestingly, higher functionality of MR in humans has been related to higher dispositional optimism, fewer thoughts of hopelessness and a lower risk on major depression (Kuningas et al., 2007; Klok et al., 2011). Yet, this effect was only observed in women (and not men) who display a haplotype related to high MR expression.

Translating these findings from humans into rodent models, we expected MR overexpression in female mice to reduce anxiety-like behavior, increase fear memory formation and context-depend memory formation. However, we report that MR-tg are highly comparable to their control littermates with regard to anxiety-like behavior, contextual memory formation as well as contextual and cued fear learning, at least in the paradigms we employed in this study.

### Characteristics of MR Overexpression in Female Mice

To examine the role of MRs in anxiety and memory formation we used transgenic mice with forebrain specific overexpression of human MR under the control of a calcium/calmodulin-dependent protein kinase II alpha (CaMKIIα) promoter (Lai et al., 2007). Lai et al. (2007) verified the increased MR mRNA levels and reported a 3–4 folds MR mRNA increase in the hippocampus and 8-fold increase in amygdala.

Female mice secrete larger amounts of corticosterone than male animals, both under basal conditions as well as after stress-exposure (Kitay, 1961; Critchlow et al., 1963; Figueiredo et al., 2002; Kitraki et al., 2004; ter Horst et al., 2012). In agreement, we found high levels of basal plasma corticosterone levels in our wild type littermates. Female mice with transgenic overexpression of MRs in the forebrain displayed a tendency towards reduced basal corticosterone levels when compared to wild types although this did not reach significance, perhaps due to the large variation observed especially in the MR-tg animals. This suggests that MR overexpression possibly causes a compensatory down-regulation of corticosterone levels. If so, this potentially stabilizes anxiety and conditioned-fear levels in female animals, since these parameters have been reported to depend on circulating corticosterone levels, at least in male rodents (see e.g., Pugh et al., 1997). These findings on corticosterone levels in females only partially support earlier findings in male mice, i.e., that forebrain-specific genetic modifications resulting in altered MR expression do not consistently affect basal corticosterone levels (Berger et al., 2006; Lai et al., 2007).

### Unconditioned Anxiety

Our data show that the forebrain-specific overexpression of MR in female mice has no effect on general anxiety-like behavior as tested in the elevated plus maze. MR-tg and control littermates spent comparable time in the open arms, and had a similar locomotor activity. This does not seem to be specific for female MR-tg mice, since we also observed comparable anxiety-like behavior in the same line of male MR-tg mice and their littermates (Kanatsou et al., unpublished observation). Two earlier studies did report that MR overexpression, in males, reduced anxiety-like behavior in the open field (Lai et al., 2007) or elevated plus maze (Rozeboom et al., 2007). This suggests that sex-dependent differences e.g., in brain circuits related to anxiety behavior could possibly explain the disparity between the earlier and our current observations. Yet, Rozeboom et al. (2007) also reported reduced anxiety-like behavior in female MR-tg mice, as determined in the elevated plus maze, in a highly comparable paradigm as we presently used. It should be pointed out that we took the cycle stage into account, which supposedly was not done in the earlier study (Rozeboom et al., 2007); this may have leveled out putative effects of MR overexpression in our study. In addition, methodological differences between the current study and earlier studies, such as the type of genetic modification, the age of the animals or the type of tests used to assess anxiety, may have contributed to the differences. For instance, we used 3 months old female mice while in earlier studies either age was not reported or animals were tested at a much older age (4–7 months), when phenotypes may have become more prominent (Berger et al., 2006; Lai et al., 2007; Rozeboom et al., 2007). We conducted *post hoc* a power analysis to determine optimal sample size to assure an adequate power to detect statistical significance. Based on this analysis, a large number of female mice (>60) would be required to reach statistical significant differences between the MR-tg and control mice. Therefore, we tentatively conclude that the current experimental conditions do not support a reduction of anxiety in female MR overexpressing mice.

### Fear Conditioning of Context and Cue

In contextual and cue fear conditioning, MR-tg female mice displayed comparable levels of freezing when compared to control animals. Studies in male animals reported that MR blockade impairs contextual (but not cued) fear memory (Zhou et al., 2010) while MR-overexpression enhances contextual fear (Kanatsou et al., unpublished observation). One possible explanation for the lack of effect in females might be that freezing had reached a ceiling, preventing a potential enhancement of contextual and cued memories by overexpression of MRs to be discernable. Interestingly, freezing levels in male MR-tg and wildtype mice were overall lower than in females (Kanatsou et al., unpublished observation), which indirectly supports the ceiling effect explanation. MR overexpression also did not affect fear memory (expressed by freezing) in a combined cue and context fear conditioning paradigm which tests the ability of animals to discriminate between a highly fearful cue-on and the “more safe” situation of cue-off. Therefore, we conclude that also the discriminative ability is not affected by overexpression of MR in female mice.

### Memory in a Non-Aversive Context

Pharmacological interventions and transgenic mouse models reducing or blocking the function of MR demonstrated impaired spatial memory in male individuals while non-spatial memory appeared to be intact (Yau et al., 1999; Berger et al., 2006). MR-deficient female mice were earlier reported to have impaired spatial as well as impaired stimulus-response strategies while MR overexpressing females showed improved spatial performance but no changes with respect to stimulus-response behavior (Arp et al., 2014). The latter might be explained by the fact that control littermates of MR-tg mice performed extremely well in the stimulus-response task, preventing further improvement in MR-tg mice (Arp et al., 2014). Here we report that MR overexpression did not affect memory formation in a non-aversive contextual learning task. Also here possible differences could have remained unnoticed due to a potential ceiling effect. This explanation, however, does not seem likely, given the DI-values in control mice, which were significantly but not dramatically above chance level.

## Conclusion

Taken together, testing female mice with forebrain-specific MR overexpression in several behavioral tasks revealed no effect on unconditioned anxiety, fear memory, the ability to discriminate between the threatening cue and the relatively safe cue-off period, and non-aversive contextual memory formation. Although we cannot exclude that effects of MR overexpression may be apparent in some of the tasks under different testing conditions, the current data suggest that MR overexpression does not substantially alter performance of female mice in these behavioral domains. This might suggest that lack in function of MRs, rather than enhanced MR function, results in clear behavioral phenotypes (Berger et al., 2006; Zhou et al., 2010; ter Horst et al., 2012, 2013).

## Author Contributions

Authors have made substantial contributions to the following: Conception and design of the study: SK, HJK, MJ. Interpretation of data: SK, MSO, APH, HJK, MJ, JRS. Acquisition of data: SK, LEK, MA, HJK. Analysis of data: SK, LEK, MA. Drafting the article critically for important intellectual content: SK, MSO, APH, JRS, MJ, HJK. Final approval of the version to be submitted: SK, LEK, MA, MSO, APH, JRS, HJK, MJ. Agreement to be accountable for all aspects of the work in ensuring that questions related to the accuracy or integrity of any part of the work are appropriately investigated and resolved: SK, LEK, MA, MSO, APH, JRS, HJK, MJ.

## Conflict of Interest Statement

The authors declare that the research was conducted in the absence of any commercial or financial relationships that could be construed as a potential conflict of interest.

## References

[B1] Akkerman S. Blokland A. Reneerkens O. van Goethem N. P. Bollen E. Gijselaers H. J.. (2012). Object recognition testing: methodological considerations on exploration and discrimination measures. Behav. Brain Res. 232, 335–347. 10.1016/j.bbr.2012.03.022 22490364

[B2] Arp J. M. ter Horst J. P. Kanatsou S. Fernández G. Joëls M. Krugers H. J.. (2014). Mineralocorticoid receptors guide spatial and stimulus-response learning in mice. PLoS One 9:e86236. 10.1371/journal.pone.0086236 24465979 PMC3897662

[B3] Balderas I. Rodriguez-Ortiz C. J. Salgado-Tonda P. Chavez-Hurtado J. McGaugh J. L. Bermudez-Rattoni F. (2008). The consolidation of object and context recognition memory involve different regions of the temporal lobe. Learn. Mem. 15, 618–624. 10.3410/f.1120613.576828 18723431 PMC2632790

[B4] Barsegyan A. McGaugh J. L. Roozendaal B. (2014). Noradrenergic activation of the basolateral amygdala modulates the consolidation of object-in-context recognition memory. Front. Behav. Neurosci. 8:160. 10.3389/fnbeh.2014.00160 24847228 PMC4021114

[B5] Berger S. Wolfer D. P. Selbach O. Alter H. Erdmann G. Reichardt H. M.. (2006). Loss of the limbic mineralocorticoid receptor impairs behavioral plasticity. Proc. Natl. Acad. Sci. U S A 103, 195–200. 10.1073/pnas.0503878102 16368758 PMC1324975

[B6] Brinks V. Berger S. Gass P. de Kloet E. R. Oitzl M. S. (2009). Mineralocorticoid receptors in control of emotional arousal and fear memory. Horm. Behav. 56, 232–238. 10.1016/j.yhbeh.2009.05.003 19447109

[B7] Brinks V. de Kloet E. R. Oitzl M. S. (2008). Strain specific fear behavior and glucocorticoid response to aversive events: modeling PTSD in mice. Prog. Brain Res. 167, 257–261. 10.1016/s0079-6123(07)67019-8 18037021

[B8] Critchlow V. Liebelt R. A. Bar-Sela M. Mountcastle W. Lipscomb H. S. (1963). Sex difference in resting pituitary-adrenal function in the rat. Am. J. Physiol. 205, 807–815. 4291060 10.1152/ajplegacy.1963.205.5.807

[B9] de Kloet E. R. Joëls M. Holsboer F. (2005). Stress and the brain: from adaptation to disease. Nat. Rev. Neurosci. 6, 463–475. 10.1038/nrn1683 15891777

[B10] de Kloet E. R. Oitzl M. S. Joëls M. (1999). Stress and cognition: are corticosteroids good or bad guys? Trends Neurosci. 22, 422–426. 10.1016/s0166-2236(99)01438-1 10481183

[B11] DeRijk R. H. Wüst S. Meijer O. C. Zennaro M. C. Federenko I. S. Hellhammer D. H.. (2006). A common polymorphism in the mineralocorticoid receptor modulates stress responsiveness. J. Clin. Endocrinol. Metab. 91, 5083–5089. 10.1210/jc.2006-0915 17018659

[B12] Di S. Malcher-Lopes R. Halmos K. C. Tasker J. G. (2003). Nongenomic glucocorticoid inhibition via endocannabinoid release in the hypothalamus: a fast feedback mechanism. J. Neurosci. 23, 4850–4857. 12832507 10.1523/JNEUROSCI.23-12-04850.2003 PMC6741208

[B13] Dix S. L. Aggleton J. P. (1999). Extending the spontaneous preference test of recognition: evidence of object-location and object-context recognition. Behav. Brain Res. 99, 191–200. 10.1016/s0166-4328(98)00079-5 10512585

[B14] Eacott M. J. Norman G. (2004). Integrated memory for object, place and context in rats: a possible model of episodic-like memory? J. Neurosci. 24, 1948–1953. 10.1523/jneurosci.2975-03.2004 14985436 PMC6730393

[B16] Figueiredo H. F. Dolgas C. M. Herman J. P. (2002). Stress activation of cortex and hippocampus is modulated by sex and stage of estrus. Endocrinology 143, 2534–2540. 10.1210/en.143.7.2534 12072385

[B17] Groc L. Choquet D. Chaouloff F. (2008). The stress hormone corticosterone conditions AMPAR surface trafficking and synaptic potentiation. Nat. Neurosci. 11, 868–870. 10.1038/nn.2150 18622402

[B18] Groeneweg F. L. Karst H. de Kloet E. R. Joëls M. (2011). Rapid non-genomic effects of corticosteroids and their role in the central stress response. J. Endocrinol. 209, 153–167. 10.1530/JOE-10-0472 21357682

[B20] Joels M. Baram T. Z. (2009). The neuro-symphony of stress. Nat. Rev. Neurosci. 10, 459–466. 10.1038/nrn2632 19339973 PMC2844123

[B22] Karst H. Berger S. Erdmann G. Schütz G. Joëls M. (2010). Metaplasticity of amygdalar responses to the stress hormone corticosterone. Proc. Natl. Acad. Sci. U S A 107, 14449–14454. 10.1073/pnas.0914381107 20663957 PMC2922581

[B23] Karst H. Berger S. Turiault M. Tronche F. Schütz G. Joëls M. (2005). Mineralocorticoid receptors are indispensable for nongenomic modulation of hippocampal glutamate transmission by corticosterone. Proc. Natl. Acad. Sci. U S A 102, 19204–19207. 10.1073/pnas.0507572102 16361444 PMC1323174

[B24] Kitay J. I. (1961). Sex differences in adrenal cortical secretion in the rat. Endocrinology 68, 818–824. 10.1210/endo-68-5-818 13756461

[B25] Kitraki E. Kremmyda O. Youlatos D. Alexis M. Kittas C. (2004). Spatial performance and corticosteroid receptor status in the 21-day restraint stress paradigm. Ann. N. Y. Acad. Sci. 1018, 323–327. 10.1196/annals.1296.039 15240385

[B26] Klok M. D. Alt S. R. Irurzun Lafitte A. J. Turner J. D. Lakke E. A. Huitinga I.. (2011). Decreased expression of mineralocorticoid receptor mRNA and its splice variants in postmortem brain regions of patients with major depressive disorder. J. Psychiatr. Res. 45, 871–878. 10.1016/j.jpsychires.2010.12.002 21195417

[B150] Kuningas M. de Rijk R. H. Westendorp R. G. Jolles J. Slagboom P. E. van Heemst D. (2007). Mental performance in old age dependent on cortisol and genetic variance in the mineralocorticoid and glucocorticoid receptors. Neuropsychopharmacology 32, 1295–1301. 10.1038/sj.npp1301260 17133261

[B28] Lai M. Horsburgh K. Bae S. E. Carter R. N. Stenvers D. J. Fowler J. H.. (2007). Forebrain mineralocorticoid receptor overexpression enhances memory, reduces anxiety and attenuates neuronal loss in cerebral ischaemia. Eur. J. Neurosci. 25, 1832–1842. 10.1111/j.1460-9568.2007.05427.x 17432969

[B29] Mumby D. G. Gaskin S. Glenn M. J. Schramek T. E. Lehmann H. (2002). Hippocampal damage and exploratory preferences in rats: memory for objects, places and contexts. Learn. Mem. 9, 49–57. 10.1101/lm.41302 11992015 PMC155935

[B30] O’Brien N. Lehmann H. Lecluse V. Mumby D. G. (2006). Enhanced context-dependency of object recognition in rats with hippocampal lesions. Behav. Brain Res. 170, 156–162. 10.1016/j.bbr.2006.02.008 16580742

[B31] Otte C. Wingenfeld K. Kuehl L. K. Kaczmarczyk M. Richter S. Quante A.. (2015). Mineralocorticoid receptor stimulation improves cognitive function and decreases cortisol secretion in depressed patients and healthy individuals. Neuropsychopharmacology 40, 386–393. 10.1038/npp.2014.181 25035081 PMC4443950

[B32] Pugh C. R. Tremblay D. Fleshner M. Rudy J. W. (1997). A selective role for corticosterone in contextual-fear conditioning. Behav. Neurosci. 111, 503–511. 10.1037//0735-7044.111.3.503 9189265

[B33] Reul J. M. de Kloet E. R. (1985). Two receptor systems for corticosterone in rat brain: microdistribution and differential occupation. Endocrinology 117, 2505–2511. 10.1210/endo-117-6-2505 2998738

[B34] Rozeboom A. M. Akil H. Seasholtz A. F. (2007). Mineralocorticoid receptor overexpression in forebrain decreases anxiety-like behavior and alters the stress response in mice. Proc. Natl. Acad. Sci. U S A 104, 4688–4693. 10.1073/pnas.0606067104 17360585 PMC1838662

[B35] Schwabe L. Schächinger H. de Kloet E. R. Oitzl M. S. (2010). Corticosteroids operate as a switch between memory systems. J. Cogn. Neurosci. 22, 1362–1372. 10.1162/jocn.2009.21278 19445601

[B36] Schwabe L. Tegenthoff M. Höffken O. Wolf O. T. (2013). Mineralocorticoid receptor blockade prevents stress-induced modulation of multiple memory systems in the human brain. Biol. Psychiatry 74, 801–808. 10.1016/j.biopsych.2013.06.001 23871473

[B37] Spanswick S. C. Dyck R. H. (2012). Object/context specific memory deficits following medial frontal cortex damage in mice. PLoS One 7:e43698. 10.1371/journal.pone.0043698 22928019 PMC3424291

[B38] Spanswick S. C. Sutherland R. J. (2010). Object/context-specific memory deficits associated with loss of hippocampal granule cells after adrenalectomy in rats. Learn. Mem. 17, 241–245. 10.1101/lm.1746710 20410060 PMC2893217

[B39] ter Horst J. P. Kentrop J. Arp M. Hubens C. J. de Kloet E. R. Oitzl M. S. (2013). Spatial learning of female mice: a role of the mineralocorticoid receptor during stress and the estrous cycle. Front. Behav. Neurosci. 7:56. 10.3389/fnbeh.2013.00056 23754993 PMC3667238

[B41] ter Horst J. P. van der Mark M. H. Arp M. Berger S. de Kloet E. R. Oitzl M. S. (2012). Stress or no stress: mineralocorticoid receptors in the forebrain regulate behavioral adaptation. Neurobiol. Learn. Mem. 98, 33–40. 10.1016/j.nlm.2012.04.006 22543192

[B42] van Leeuwen N. Bellingrath S. de Kloet E. R. Zitman F. G. DeRijk R. H. Kudielka B. M.. (2011). Human Mineralocorticoid Receptor (MR) gene haplotypes modulate MR expression and transactivation: implication for the stress response. Psychoneuroendocrinology 36, 699–709. 10.1016/j.psyneuen.2010.10.003 21095064

[B43] Yau J. L. Noble J. Seckl J. R. (1999). Continuous blockade of brain mineralocorticoid receptors impairs spatial learning in rats. Neurosci. Lett. 277, 45–48. 10.1016/s0304-3940(99)00858-7 10643894

[B44] Zhou M. Bakker E. H. Velzing E. H. Berger S. Oitzl M. Joëls M.. (2010). Both mineralocorticoid and glucocorticoid receptors regulate emotional memory in mice. Neurobiol. Learn. Mem. 94, 530–537. 10.1016/j.nlm.2010.09.005 20849967

[B45] Zhou M. Conboy L. Sandi C. Joëls M. Krugers H. J. (2009). Fear conditioning enhances spontaneous AMPA receptor-mediated synaptic transmission in mouse hippocampal CA1 area. Eur. J. Neurosci. 30, 1559–1564. 10.1111/j.1460-9568.2009.06951.x 19811531

[B46] Zhou M. Kindt M. Joëls M. Krugers H. J. (2011). Blocking mineralocorticoid receptors prior to retrieval reduces contextual fear memory in mice. PLoS One 6:e26220. 10.1371/journal.pone.0026220 22022574 PMC3192177

